# Targeting Myostatin for Sustainable Meat Production: Insights From Multiple Species

**DOI:** 10.1002/age.70149

**Published:** 2026-06-14

**Authors:** Chiara Santomassimo, Francesco Perini, Daniele Colombi, Stefano Bettini, Maria Federica Trombetta, Marina Pasquini, Simone Ceccobelli, Ishaku L. Haruna, Ketan Patel, Emiliano Lasagna

**Affiliations:** ^1^ Department of Agricultural, Food and Environmental Sciences University of Perugia Perugia Italy; ^2^ Department of Chemistry, Biology and Biotechnology University of Perugia Perugia Italy; ^3^ National Association of Italian Beef‐Cattle Breeders (ANABIC) Perugia Italy; ^4^ Department of Agricultural, Food and Environmental Sciences Università Politecnica delle Marche Ancona Italy; ^5^ Department of Biology Gombe State University Gombe Nigeria; ^6^ School of Biological Science University of Reading Berkshire UK

**Keywords:** double muscling, feed conversion, meat production, myostatin, reproduction

## Abstract

Myostatin gene (*MSTN*), a TGF‐β superfamily member, negatively regulates skeletal muscle growth, positioning it as a prime genetic target for enhancing livestock meat production. This review synthesizes evidence from 1979 to 2025 on *MSTN* mutations across cattle, pigs, poultry, sheep, goats, and rabbits, emphasising impacts on yield, quality, reproduction, and feed efficiency. Loss‐of‐function variants drive 20%–30% muscle mass increases in double‐muscled phenotypes, boosting lean yield, carcass conformation, and dressing percentages while reducing fat (decreased by 50%). These changes improve growth rates and feed conversion ratios, and overall sustainability by lowering resource inputs and emissions, aligning with demands for low‐fat and high‐quality protein. Reproduction exhibits species‐specific challenges, such as reduced fertility or dystocia, which are often manageable in heterozygotes through targeted breeding, while meat quality benefits from tender, lean profiles. From natural alleles to CRISPR/Cas9 knockouts in goats/pigs, *MSTN* manipulation enhances productivity without major welfare trade‐offs. Multi‐species insights underscore the value of heterozygous strategies for balanced gains by integrating genomic selection and editing tools to meet rising global meat needs efficiently, resiliently, and ethically.

## 1 Introduction

### 1.1 Brief Overview of Global Meat Consumption and the Role of Animal Production in Food Security

The global demand for high‐quality meat has grown significantly, driven by consumer preferences for products with enhanced nutritional value, superior tenderness, rich flavour, and environmentally sustainable production, as indicated by Santos et al. (2021). Meat production, especially beef, pork, and poultry, remains the dominant contributor to global protein supply, although production trends vary significantly across regions due to complex economic, structural, and environmental factors (FAO 2024; ISMEA 2024). In many countries, persistent structural limitations and a heavy reliance on imported meat underline the urgent need for innovation and greater self‐sufficiency in the livestock sector (FAO 2024).

Addressing the closely interconnected diet–environment–health trilemma through dietary solutions represents both a global challenge and a unique opportunity, with deep implications for environmental sustainability and public health (Tilman and Clark 2014).

**FIGURE 1:**
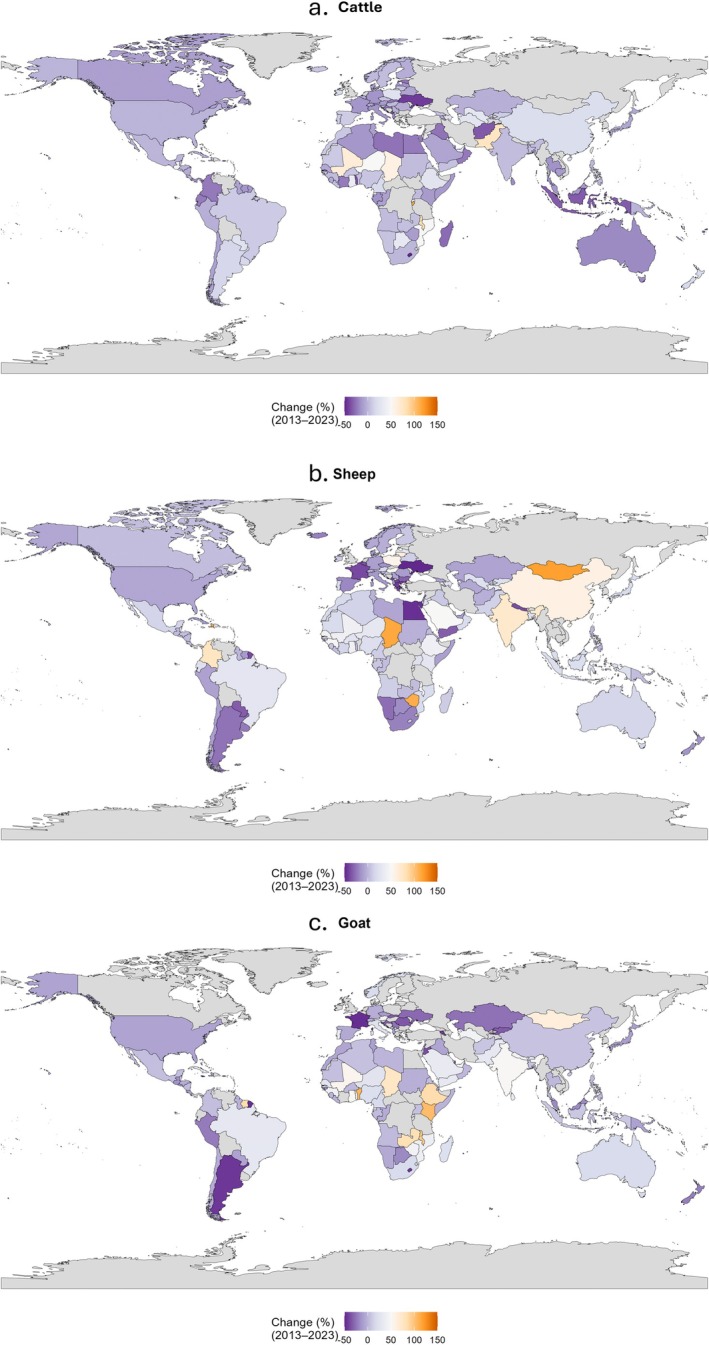
Comparison of global meat production (tonnes) in ruminants between 2013 and 2023 based on FAOSTAT data: Species (from a–c) and country level variations highlighting trends and structural shifts in livestock systems.

**FIGURE 2:**
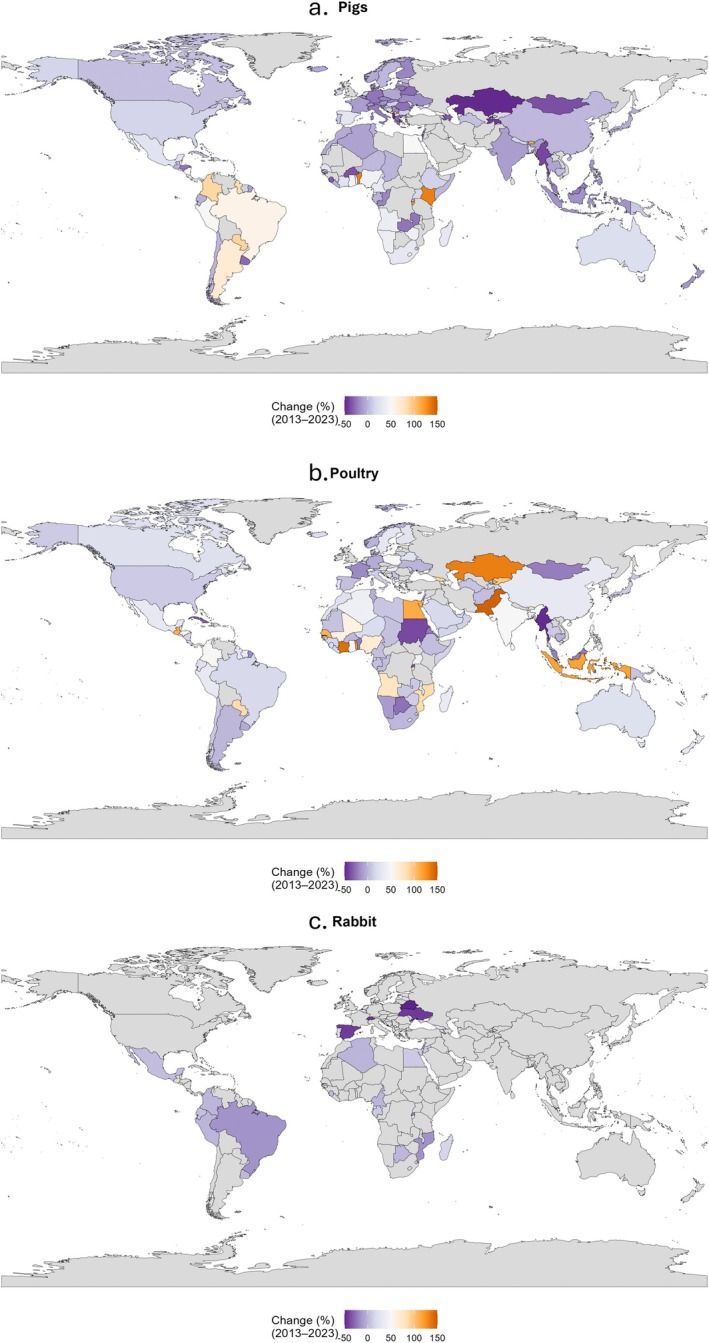
Comparison of global meat production (tonnes) in non‐ruminants between 2013 and 2023 based on FAOSTAT data: Species (from a–c) and country level variations highlighting trends and structural shifts in livestock systems.

Analysis of FAOSTAT data over the last decade highlights substantial changes in meat production across livestock species and geographical regions worldwide. Between 2013 and 2023, global meat production exhibited differing trends across species and geographic areas, with marked increases in many middle‐income countries and declines or stagnation in several mature economies. Figures 1 and 2 reveal substantial data gaps across several countries, particularly regarding rabbit production and certain African regions, pointing to structural limitations in statistical systems and production flows traceability. In the cattle beef sector, numerous countries in South America, Oceania, and parts of Europe recorded reductions or very modest growth, whereas several areas in Central Asia and the Middle East experienced significant percentage increases, consistent with an expansion of cattle production systems in emerging regions (Figure 1a). In contrast, poultry production (Figure 2b) showed widespread growth; many countries in Africa, the Middle East, and Asia reported increases often exceeding 50%, with only a few localized declines. These trends confirm the role of poultry as the fastest‐expanding source of meat worldwide. Pork production (Figure 2a) tended to decline or grow only modestly in various Asian and European countries, potentially reflecting the impact of sanitary crises and increasingly stringent environmental regulations. In contrast, several South American and African countries exhibited strong growth, indicating a partial geographic reallocation of the sector. Sheep and goat production (Figure 1b,c) increased markedly in several African and Latin American countries but decreased in some major Asian and Oceanian nations, underscoring the adaptability of small ruminants to marginal agro‐climatic environments. Lastly, rabbit meat production was highly concentrated (Figure 2b). Notable increases were observed only in a few countries in Eastern Europe, China, and South America, whereas extensive areas worldwide lacked available data, confirming the niche or poorly monitored nature of this livestock sector at the global level (FAO 2024).

The variations observed in global meat production reflect a combination of economic, environmental and social drivers. In many emerging economies, including nations like Kazakhstan, output growth is largely associated with population increase, rising per capita income, and the resulting shift in dietary preferences towards animal‐source foods (Komarek et al. 2021; van der Laan et al. 2024). At the same time, government policies that support livestock sector modernisation, investment in intensive production systems, and genetic improvement have further contributed to production expansion in several regions (FAO 2024; Vernooij and van Mierlo 2021). By contrast, marked reductions in meat production in countries such as Australia, India and Brazil are linked to recurrent climatic extremes (droughts and wildfires), the impact of severe epidemic diseases such as African swine fever, and the implementation of stricter environmental regulations aimed at limiting greenhouse gas emissions (Brown et al. 2021; Silveira et al. 2025; van der Laan et al. 2024). In addition, socio‐economic crises and political instability in some regions have undermined the productive capacity of the livestock sector and exacerbated the declines detected by the FAO reports (Mehrabi et al. 2020).

Ensuring access to high‐quality protein sources for a rapidly growing global population remains a major challenge for both the scientific community and policymakers. Sustainable, scalable, and economically viable production strategies will be essential to safeguard global food security. Such sources must be not only nutritionally adequate but also derived from sustainable production systems capable of reducing environmental impact and preserving natural resources. In this context, skeletal muscle development plays a crucial role as the biological precursor of meat, the main source of high‐quality animal protein for human consumption. The quantity and quality of muscle growth in livestock directly influence carcass yield as well as the nutritional and sensory properties of meat after slaughter. Consequently, a comprehensive understanding of the genetic and physiological mechanisms involved in muscle development is essential for enhancing both productive efficiency and meat quality.

Among the various genes involved in muscle development, one of the most extensively investigated and functionally significant is the myostatin gene (*MSTN*), a well‐known negative regulator of muscle growth. The inactivation or mutation of this gene has been associated with increased muscle mass (Casas et al. 2004), enhanced meat quality (Picard and Gagaoua 2020), and potential benefits for animal welfare (Lee et al. 2024), with positive impacts on overall production efficiency and the sustainability of livestock systems (Kalds et al. 2023; Table 1). Nonetheless, *MSTN* mutations have also been linked to reduced reproductive efficiency (Wiener et al. 2002).

**TABLE 1:** Overall effects of *MSTN* inactivation categorized by ruminant and non‐ruminant species.

Species	Effects on muscle growth/meat production	Effects on reproductive traits	Effects on feed conversion	Considerations for meat quality	References
Cattle	Double‐muscling phenotype (Piemontese, Marchigiana); ↑ carcass yield (up to +25% muscle mass), ↓ fat	↑ dystocia, often requiring caesareans; heterozygotes maintain adequate fertility	↑ feed efficiency, ↑ ADG, ↓ FCR	↓ marbling and flavour in some breeds; improved tenderness due to ↓ collagen	Ceccobelli et al. (2022); Kalds et al. (2023); Kobolák and Gócza (2002); Ryan et al. (2023)
Sheep	Texel: natural double muscling; ↑ hypertrophy and carcass yield (up to +20%–25% muscle mass)	↑ dystocia due to higher birth weights; gene‐edited sheep show limited reproductive effects but require monitoring	↑ feed efficiency, ↑ growth with the same diet	Tendency for ↓ marbling, but tenderness is maintained in *MSTN*‐KO lambs	Clop et al. (2006); Haynes et al. (2012); Tellam et al. (2012)
Goats	*MSTN*‐KO and *MSTN* polymorphisms ↑ affect body weight and carcass yield.	Potential birthing complications and skeletal development issues; limited data on adult fertility.	Likely ↑ feed efficiency due to ↑ muscle and ↓ fat. ↑ ADG of 36%	Possible changes in marbling and lipid profiles; important for breeding plans	Kalds et al. (2023); Wang et al. (2018); Zhang et al. (2012)
Pigs	*MSTN*‐KO: ↑ muscle, ↓ backfat; heterozygotes improve carcass yield (up to +23.77% muscle mass)	Delayed puberty, ↓ litter size, ↑ congenital defects (macroglossia, hernias)	↑ nutrient partitioning efficiency towards muscle	↓ intramuscular fat reduces taste and tenderness; combining with other markers is suggested	Gao (2024); Han et al. (2021); Kalds et al. (2023); Qian et al. (2015)
Poultry	↑ muscle mass, ↓ fat; *MSTN*‐KO chickens and quail are heavier (up to +15%–30% muscle mass)	Delayed egg laying onset, ↓ fertility and hatchability, ↑ egg weight	↓ FCR, ↑ efficiency, and better nutrient utilisation	↓ intramuscular fat may reduce juiciness and flavour	Lee et al. (2024), Lee et al. (2021b); Shoyombo et al. (2022)
Rabbits	*MSTN*‐KO: ↑ muscle mass (+20%–30% BW), with hyperplasia and hypertrophy	Skeletal deformities (pelvic tilt) can impair mating and parturition	↓ *MSTN* expression is associated with ↑ feed efficiency	Impact on tenderness, juiciness, and muscle fibre types needs further evaluation	Kuang et al. (2014); Sternstein et al. (2014); Zhang, Lu, et al. (2019); Zhang, Ran, et al. (2019)

Meat quality shows substantial variation both across different muscles and among individual animals, primarily influenced by variations in metabolic and contractile properties determined by muscle fibre type composition. Such variability arises from a complex interplay between intrinsic (genetic) and extrinsic (environmental) factors that affect metabolic processes during both the peri‐ and post‐mortem phases (Klont et al. 1998). Meat quality remains a major challenge for global markets, as its key traits are generally measurable only post‐mortem, through expensive and time‐consuming analytical procedures, and often exhibit moderate to low heritability (Boukha et al. 2011). Genetic components, particularly heritable traits, play a fundamental role in determining meat quality (Kobolák and Gócza 2002). Intrinsic attributes such as tenderness, juiciness, flavour, colour, fat distribution, and nutrient composition, along with extrinsic factors like husbandry practices and price, are crucial in shaping consumer perception and beef marketability, thereby representing traits of significant economic importance for the industry (Romero et al. 2024). Recent advancements in genomic technologies have revolutionised livestock breeding by enabling more accurate selection for desirable phenotypes. Single nucleotide polymorphisms (SNPs) markers are instrumental in identifying candidate genes associated with economically important traits. Genomic selection strategies based on these markers have facilitated the development of beef products with enhanced quality attributes aligned with consumer preferences (Romero et al. 2024).

This review was based on a comprehensive literature survey investigating the role of *MSTN* in livestock species, with particular emphasis on muscle growth, meat quality, reproductive performance, and feed efficiency (FE). The scientific literature reviewed in this article was selected primarily from the Scopus, PubMed, and Google Scholar databases. The research included peer‐reviewed journal articles, reviews, and book chapters using a combination of keywords such as *“myostatin”, “MSTN mutation”, “double muscling”, “livestock”, “cattle”, “sheep”, “goat”, “pig”, “poultry”, “rabbit”, “meat quality”, “feed efficiency”, “reproduction”, and considered studies published between 1979 and 2025*. Studies that directly addressed *MSTN* function, genetic variants, or gene editing applications in livestock species were included. Moreover, articles focusing exclusively on human or laboratory animal models were excluded unless they provided mechanistic insights directly translatable to livestock. When multiple studies reported similar findings, preference was given to the most recent or comprehensive analyses.

### 1.2 Genetic and Physiological Importance of Myostatin as a Negative Regulator of Muscle Growth

Myostatin (MSTN), also known as growth and differentiation factor 8 (GDF8), is a member of the transforming growth factor‐beta (TGF‐β) superfamily serving as a key negative regulator of skeletal muscle development across mammalian species (Aiello et al. 2018). MSTN inhibits muscle growth by controlling myoblast proliferation and differentiation during both prenatal and postnatal stages, ensuring the regulation of overall muscle mass (Jouliaekaza and Cabello 2007). Loss‐of‐function mutations in *MSTN* lead to a remarkable increase in skeletal muscle mass through the combined effect of hyperplasia, defined as an increase in muscle fibre number, and hypertrophy, defined as an enlargement of fibre cross‐sectional area. This phenotype is commonly referred to as “double muscling” (DBM) (Bellinge et al. 2005; McPherron et al. 1997). This trait has been documented in several livestock species, including cattle (e.g., Belgian Blue, Piemontese), sheep (e.g., Texel), and even rabbits and pigs (Aiello et al. 2018; Chen et al. 2021). DBM animals exhibit superior carcass yields, reduced fat deposition, and improved proportions of high‐value meat cuts, meeting growing consumer demands for leaner and higher‐quality products (Bellinge et al. 2005). These phenotypic changes are not only biologically relevant but also economically advantageous for meat production. In particular, animals carrying *MSTN*‐inactivating alleles can produce up to 20%–30% more muscle mass and approximately 50% less fat than their wild‐type counterparts, resulting in improved production efficiency and leaner meat that better meets current nutritional recommendations and consumer preferences (Aiello et al. 2018). Moreover, the increased proportion of fast‐twitch glycolytic fibres in DBM animals results in tender meat, although careful management is required to optimise intramuscular fat levels and flavour (Smet et al. 2000). In addition to quantitative benefits, *MSTN*‐mutated animals may contribute to more sustainable production systems. Enhanced FE and greater muscle growth relative to maintenance costs could lead to reduced resource inputs (feed and water) (Lee et al. 2021a) and lower greenhouse gas emissions, addressing critical environmental concerns associated with livestock farming (Mckimmie et al. 2022). New biotechnological tools, such as CRISPR/Cas9 gene editing, now allow precise *MSTN* editing without introducing exogenous DNA. This approach enables the creation of *MSTN*‐null animals with improved production traits while potentially mitigating animal welfare concerns through targeted breeding programs (Zhou et al. 2022).

Collectively, these findings indicate that *MSTN* variation may constitute a key component of future breeding strategies aimed at meeting the increasing global demand for high‐quality meat while minimising environmental and economic impacts. Accordingly, a comprehensive understanding of the mechanisms of MSTN regulation and its physiological effects provides a robust framework for exploring its role in productivity, reproductive performance, and feed conversion efficiency across livestock species, as discussed in the following sections, which follow the order of species presented in Figures 1 and 2.

### 1.3 The Regulatory Mechanisms of Myostatin in Muscle Growth

MSTN negatively regulates myogenesis through autocrine and paracrine signalling mediated by activin type II‐A and II‐B receptors (Baig et al. 2022; Chen et al. 2021). Upon ligand binding, MSTN activates the canonical Smad2/3 signalling pathway, leading to transcriptional repression of key myogenic regulatory factors, including *MYOD* and *MYOG*, thereby inhibiting myoblast proliferation and differentiation (Chen et al. 2021). As a result, MSTN limits muscle hyperplasia during prenatal development by restraining myoblast expansion. In postnatal muscle, MSTN suppresses hypertrophy by modulating protein turnover through both inhibition of anabolic processes and activation of catabolic pathways. Specifically, MSTN interferes with anabolic signalling pathways, reducing protein synthesis, while concurrently promoting protein degradation via FOXO‐mediated upregulation of E3 ubiquitin ligases, such as Atrogin‐1/MAFbx and MuRF1. This activation enhances ubiquitin–proteasome‐mediated proteolysis and autophagy, contributing to muscle atrophy (Baig et al. 2022; Saneyasu et al. 2019). Beyond the canonical pathway, MSTN also modulates several non‐canonical signalling cascades, including WNT4/β‐catenin and MAPK pathways (Erk1/2, JNK, and p38), which further fine‐tune myoblast activity, differentiation, and overall muscle homeostasis (Steelman et al. 2006).

At the extracellular level, MSTN activity is tightly regulated through proteolytic processing and binding interactions. The precursor pro‐MSTN is activated by proteases such as furin and BMP‐1/Tolloid, while its bioavailability is controlled by inhibitory binding proteins, including follistatin, which prevents MSTN interaction with ActRIIB and subsequent Smad phosphorylation (Amthor et al. 2004).

Consistently, loss‐of‐function mutations in *MSTN* result in a marked increase in muscle mass due to the combined effects of enhanced hyperplasia and hypertrophy. This “double‐muscling” phenotype has been widely observed across livestock species and has been associated with increased meat yield, although it also underscores the importance of balanced breeding strategies to avoid potential negative trade‐offs.

### 1.4 The Role of Myostatin in Meat Production and Quality

#### 1.4.1 Cattle

The myostatin gene (*MSTN*), located on chromosome 2, harbours loss‐of‐function mutations that are among the most significant genetic factors influencing muscle growth and meat production in cattle (Aiello et al. 2018). MSTN acts as a negative regulator of skeletal muscle development; its inactivation leads to the well‐known “double‐muscled” phenotype (Kobolák and Gócza 2002). This phenotype is most prominently observed in Belgian Blue (BB) (c.821del11) (Aiello et al. 2018; Fiems et al. 2020), Piemontese (c.938G>A) (Aiello et al. 2018; Boukha et al. 2011) and Marchigiana (c.871G>T) (Colombi, Perini, et al. 2024; Colombi, Rovelli, et al. 2024) breeds, but also occurs in Charolais (g.2283A>G) (Aiello et al. 2018), Limousin (c.282C>A), and other European beef breeds due to various functional *MSTN* mutations (Zhao et al. 2022). DBM cattle exhibit significant improvements in carcass yield and meat quality traits. BB cattle achieve dressing percentages exceeding 60%, representing a notable improvement compared to conventional breeds (Abdullah and Meng 2024). This enhanced carcass yield is accompanied by significant changes in meat composition: *MSTN*‐mutant cattle produce leaner meat, with intramuscular fat reduced by 30%–50% and total fat content reduced by up to 50% (Kobolák and Gócza 2002). Although *MSTN* mutations can reduce collagen content, they also increase muscle fibre number and cross‐sectional area, shift fibre composition toward fast‐twitch glycolytic fibres, and reduce intramuscular fat, which may counteract tenderness improvements (Hocquette et al. 2006). Recent advances in gene editing further confirm the potential of *MSTN*‐targeted breeding. For instance, CRISPR/Cas9 *MSTN*‐knockouts in Chinese Yellow Cattle have demonstrated that muscle growth and carcass yield can be enhanced while maintaining satisfactory levels of health and reproductive performance (Zhao et al. 2022). Similarly, crossbreeding programs that integrate BB genetics into *Bos indicus* populations under tropical environments have yielded hybrids exhibiting 50 to 100% higher live weight gains, along with improved carcass traits (Abdullah and Meng 2024). Nevertheless, the productive advantages associated with *MSTN* mutations are accompanied by important biological trade‐offs. The reduction in marbling (intramuscular fat) may compromise juiciness and flavour, key sensory attributes valued in specific consumer markets (Ceccobelli et al. 2022; Fiems et al. 2020; Romero et al. 2024). Moreover, DBM animals often show skeletal fragility due to reduced bone mass, potentially requiring targeted nutritional management, particularly with respect to calcium and phosphorus supplementation to support structural integrity (Fiems 2012).

These considerations underscore the importance of integrating *MSTN* selection with additional genetic markers related to meat quality and animal welfare, ensuring that productivity improvements align with sustainable and ethical beef production goals.

The double‐muscling phenotype results in carcasses characterised by higher dressing percentages and enhanced muscle‐to‐bone ratios, making them highly desirable for markets that prioritise low‐fat beef. Evidence from Marchigiana bulls indicates that heterozygous animals may represent an optimal compromise, showing superior carcass conformation and lean yield while maintaining desirable productive traits (Ceccobelli et al. 2022), because they were not affected in growth rates in a relevant manner while showing increased muscularity (Colombi, Perini, et al. 2024). Although *MSTN* mutations are generally associated with increased tenderness and advantageous muscle growth, their impact on technological properties is multifaceted. For instance, alterations in meat colour and water‐holding capacity have also been reported (Picard and Gagaoua 2020), requiring careful consideration during meat processing and quality assessment. Moreover, while the lower collagen content in *MSTN*‐deficient animals contributes to improved tenderness, it is essential that flavour and juiciness are not adversely affected. Future breeding programs may therefore benefit from combining *MSTN* selection with genetic markers related to fat deposition and flavour to meet both production efficiency and consumer expectations (Romero et al. 2024).

#### 1.4.2 Sheep

In sheep ( *Ovis aries* ), *MSTN* has been extensively studied. It is located on chromosome 2 at the 2q32.2 locus, near the distal end of the long arm (Aiello et al. 2018). Natural mutations, such as the well‐documented g.6723G>A SNP in Texel sheep, have been associated with enhanced muscularity through disruption of miRNA binding sites, leading to the DBM phenotype. This mutation has been linked to increased loin depth (+2.8%) and loin area (+3.2%) (Clop et al. 2006). By promoting myoblast proliferation and reducing fat deposition, this variant induces muscle hypertrophy, making Texel sheep an important model for *MSTN*‐related research. Hickford et al. (2010) investigated *MSTN* variation in New Zealand Romney sheep and identified allele B as being positively associated with loin yield and total lean meat percentage, suggesting its utility in marker‐assisted selection. Similarly, Boman et al. (2009) reported that a frameshift mutation in Norwegian White Sheep (c.960delG) significantly influenced carcass conformation and fatness, highlighting the potential of *MSTN* variations to enhance production traits across diverse sheep breeds. Recent genomic studies have further clarified the role of *MSTN* in ovine meat production. Han et al. (2025), through integrated genome‐wide association studies (GWAS) and RNA‐seq analyses, identified *MSTN* as a key candidate gene associated with muscle development, intramuscular fat content, and growth rate across different sheep populations. *MSTN* interacts with other key genes, including *LCORL* (body size regulation), *BMP2* (bone formation), *MEF2B* (muscle fibre differentiation), and *FABP4* (fat deposition), suggesting that muscle growth and development in sheep is a polygenic trait modulated by *MSTN* within a broader genetic network (Han et al. 2025). These findings highlight the importance of integrating *MSTN* with additional markers for efficient genetic improvement. Genome‐editing studies have provided further evidence of *MSTN's* functional relevance. More recently, Zhou et al. (2022) optimized CRISPR/Cas9 delivery in one‐cell ovine embryos by testing Cas9 mRNA+ sgRNA combinations, identifying Cas9 mRNA + sgRNA as optimal for ~50% targeting efficiency (25% homozygotes). *MSTN*‐knockout lambs exhibited higher birth weight (4.4 vs. 3.9 kg) and average daily gain, with no changes in meat quality traits (pH, drip loss, intramuscular fat, crude protein, shear force in gluteal muscle) or animal health compared to wild‐types. These advances underscore growing interest in *MSTN*‐targeted editing for enhanced ovine productivity.

Nevertheless, the impact of *MSTN* mutations on meat quality warrants careful evaluation. Sousa‐Junior et al. (2022) found significant associations between *MSTN* haplotypes and tenderness, pH, and water‐holding capacity in Santa Inês sheep, indicating that *MSTN* variants may influence sensory qualities of lamb meat. Hadjipavlou et al. (2008) demonstrated that certain *MSTN* polymorphisms in Charollais sheep were linked to deeper muscle profiles, which could improve carcass classification but potentially reduce intramuscular fat content. Interestingly, Zhou et al. (2022) reported no significant differences in intramuscular fat or shear force between *MSTN*‐knockout and wild‐type lambs, suggesting that *MSTN* disruption may enhance lean yield without compromising tenderness. Overall, combining *MSTN*‐based selection or genome editing with markers associated with marbling and flavour‐related traits could provide a more balanced approach, ensuring both production efficiency and consumer acceptance in modern sheep breeding programs.

#### 1.4.3 Goats

In goats, *MSTN* polymorphisms have been reported mainly in the 5′‐UTR and exon 1 on chromosome 2 (Aiello et al. 2018). *MSTN* is increasingly recognised as a key target for enhancing meat production in goats. Naturally occurring *MSTN* polymorphisms and targeted gene‐editing technologies have demonstrated significant potential in boosting growth performance and carcass traits across several caprine breeds. For instance, Dowidar et al. (2018) reported a high nucleotide similarity (up to 99%) in *MSTN* sequences from Egyptian goat breeds (Zaraibi, Baladi, and Damascus) and high‐yielding Chinese breeds (e.g., Qianbei Ma, Guizhou Black), alongside 95%–99% conservation with sheep and cattle sequences across specific introns and exons (accessions: KY463684–KY463685, KY441464‐KY463686). These findings suggest a valuable genetic resource for selection programs aimed at improving meat yield. Similarly, Zhang et al. (2012) identified two relevant *MSTN* polymorphisms (c.1256_1260del5 and c.1388 T>A) in Boer goats significantly associated with growth performance. Goats with a heterozygous AB genotype outperformed BB homozygotes in live weight by 0.3 kg at birth, 1.1 kg at 90 days, and 1.4 kg at 300 days, and also exhibited significantly greater body length and height, suggesting *MSTN* as a promising candidate for marker‐assisted selection. Beyond naturally occurring variation, advanced gene‐editing technologies have successfully allowed the creation of *MSTN*‐disrupted goats with enhanced muscle growth. Kalds et al. (2023) described CRISPR/Cas9‐mediated *MSTN*‐knockout goats exhibiting significantly higher average birth weight and body weight from Day 0 to Day 360, including the weaning phase, from Day 70 to Day 90. By the end of the experimental period, edited goats reached approximately 55 kg compared with around 40 kg in wild‐type controls. They also showed increased average daily gain (ADG), body height, length, and chest circumference/depth/width at multiple growth stages (Days 30, 60 and 90). Importantly, no adverse effects on health status or blood biochemical profile were detected. Transcriptomic analyses further revealed differential expression of genes involved in fatty acid metabolism, indicating that *MSTN* disruption exerts broader metabolic effects beyond muscle growth. Collectively, these studies confirm that targeting *MSTN* in goats can produce a DBM phenotype like that seen in other livestock species, offering a substantial increase in lean meat production.

However, while *MSTN* disruption in goats offers clear advantages for carcass yield, its effects on meat quality require careful evaluation. Zhang et al. (2012) noted that *MSTN* polymorphisms may influence fat deposition patterns, with possible consequences for marbling and tenderness. Interestingly, Kalds et al. (2023) reported that *MSTN*‐edited goats exhibited improved muscle mass without deleterious effects on FE or overall health, suggesting that gene editing could enhance meat quantity without compromising quality traits. Furthermore, transcriptomic analyses revealed differential expression in pathways linked to lipid metabolism, raising questions about potential shifts in intramuscular fat content and fatty acid profiles. These findings underscore the importance of integrating *MSTN*‐driven growth improvements with genetic selection strategies targeting meat sensory qualities and nutritional quality to ensure consumer satisfaction.

#### 1.4.4 Pigs

*MSTN* has become a key target for genetic improvement in pigs aimed at enhancing lean meat production. Polymorphisms have been reported in the promoter region, intron 1, intron 2, and exon 3 on chromosome 15 (Aiello et al. 2018). *MSTN* mutations have been shown to promote skeletal muscle hypertrophy and hyperplasia, resulting in a DBM phenotype in pigs similar to that observed in cattle and sheep (Qian et al. 2015). Using zinc‐finger nuclease (ZFN) technology, *MSTN*‐edited Meishan pigs exhibited a remarkable increase in muscle mass with reduced fat deposition, demonstrating the feasibility of applying *MSTN*‐targeted breeding to improve carcass composition in indigenous fat‐type pig breeds and involving the g.879T>A polymorphism (Aiello et al. 2018; Qian et al. 2015).

In Duroc × Meishan crossbred populations, heterozygous *MSTN*+/− pigs displayed higher dressing percentages, larger *Longissimus dorsi* muscle areas, and thinner backfat compared to wild‐type animals, indicating improved lean meat yield (Li et al. 2020). Similarly, Gao (2024) reported that homozygous *MSTN*−/− pigs showed a significant increase in carcass lean percentage and a reduction in backfat thickness. These findings highlight *MSTN* as a promising candidate for improving meat quality and production traits in commercial pig breeding programs. Marker‐assisted selection and genome editing approaches focusing on *MSTN* could enable the development of pigs with superior growth performance and FE. However, the application of *MSTN*‐based breeding strategies must carefully consider potential trade‐offs to ensure long‐term sustainability and animal welfare.

Beyond their effects on lean meat yield, *MSTN* mutations significantly influence meat quality traits in pigs. *MSTN*−/− pigs exhibit reduced intramuscular fat content, which can affect the flavour, juiciness, and overall palatability of meat (Gao 2024). Integrated transcriptomic and metabolomic analyses have revealed alterations in lipid metabolism and collagen structure in *MSTN*‐deficient pigs, both of which are critical determinants of tenderness and sensory quality (Gao 2024). Palma‐Granados et al. (2024) identified SNPs in genes such as *FASN* and *ACACA* that could be integrated with favourable *MSTN* alleles to improve intramuscular fat content and fatty acid profiles, thereby maintaining meat quality while promoting lean growth. Furthermore, Matika et al. (2019) suggested that heterozygous *MSTN* mutations may confer a balanced compromise between improved carcass composition and acceptable meat quality, while avoiding adverse effects such as leg weakness syndrome and increased postnatal mortality reported in homozygous mutants (as in the current study). Overall, combining *MSTN* gene editing with additional markers associated with quality traits could provide a holistic approach for producing pork that meets consumer expectations for both leanness and eating quality.

#### 1.4.5 Poultry

In chickens, *MSTN* is located on chromosome 7p11 and contains three exons and two introns. Most reported polymorphisms are distributed across the 5′‐and 3′‐regulatory regions, exons 1–3, and introns 1–2 (Aiello et al. 2018). *MSTN* has emerged as a promising target in poultry genetics for improving meat production efficiency. The phenomenon of muscle proliferation and differentiation following *MSTN* inhibition associated with the g.2283A>G polymorphism has been confirmed in chickens and quails, demonstrating conserved anti‐myogenic functions across both avian and mammalian species (Aiello et al. 2018; Lee et al. 2024; Shoyombo et al. 2022). Polymorphisms within *MSTN* have been associated with growth and carcass traits in broilers. For instance, Zhang, Lu, et al. (2019) and Zhang, Ran, et al. (2019) identified significant associations between *MSTN* SNPs and body weight, breast muscle yield, and carcass traits in Daheng broilers. Birds carrying favourable *MSTN* genotypes exhibited superior growth performance, suggesting that *MSTN* could serve as a candidate for marker‐assisted selection. Similarly, Bhattacharya and Chatterjee (2013) reported *MSTN* polymorphisms affecting body weight and growth rate in Indian broiler lines, further reinforcing its regulatory role in economically important traits in poultry. The advent of genome‐editing technologies, such as CRISPR/Cas9, has enabled the creation of *MSTN*‐knockout birds to further explore its functional potential more comprehensively. Although *MSTN* mutant chickens and quails exhibit increased muscle mass, reduced fat deposition, and improved FE (Lee et al. 2021b), these productive advantages are accompanied by challenges in balancing production and reproductive performance. In particular, in layer birds, *MSTN* mutations (a three‐base‐pair deletion that causes the removal of cysteine at position 42 (C42del)) have been associated with a delayed onset of egg laying and reduced egg production during the active laying phase, thereby underscoring the trade‐offs involved in using *MSTN* as a breeding target (Lee et al. 2021b). Recent studies also indicate that MSTN inactivation may enhance skeletal robustness. In Japanese quail, *MSTN* mutants showed increased tibia size, cortical thickness, and bone mineral density, potentially mitigating leg weakness issues in fast‐growing broilers (Lee et al. 2023). This combined effect on muscle accretion and bone strength highlights the relevance of MSTN in improving both poultry health and productivity.

Despite the clear benefits in muscle yield, the impact of *MSTN* mutations on meat quality remains complex. Studies suggest that MSTN inhibition in poultry results in carcasses with reduced fat content (Shoyombo et al. 2022). Although this aligns with consumer demands for low‐fat meat, poultry already contains low levels of intramuscular fat, typically below 1%, meaning that further reductions may offer limited nutritional significance and could negatively affect juiciness and flavour, key attributes for meat palatability (Zhang, Lu, et al. 2019; Zhang, Ran, et al. 2019). Moreover, modifications in muscle fibre composition in *MSTN*‐deficient birds may impact physical properties, such as water‐holding capacity and tenderness, which are key parameters for processing and storage (Bhattacharya and Chatterjee 2013). Lee et al. (2021a) highlighted that careful management and selection strategies are required to balance lean yield with sensory qualities in meat. Marker‐assisted selection integrating *MSTN* with additional loci influencing meat quality traits could provide a strategy to achieve both production efficiency and consumer satisfaction.

#### 1.4.6 Rabbits

In rabbits, *MSTN* polymorphisms have been detected in the 5′ regulatory region, exons 1–2, introns 1–2, and the 3′‐UTR on chromosome 3 (Aiello et al. 2018). *MSTN* plays a pivotal role in rabbit muscle development, as in other livestock species, and has emerged as a key target for improving meat production. Natural and experimentally induced *MSTN* variants have been investigated for their association with growth traits and carcass yield. Abdel‐Kafy et al. (2016) identified three SNPs in the rabbit *MSTN*, like c.713T>A, c.747 + 34C>T, and c.*194A>G, and demonstrated that the G allele at c.*194A>G was significantly associated with higher live body weight, average daily gain, and favourable carcass traits. Thus, these findings suggest this allele may serve as a valuable marker in breeding programs focused on growth performance. More recently, gene‐editing technologies have advanced the application of *MSTN* manipulation in rabbits. Lv et al. (2016) successfully generated *MSTN*‐knockout rabbits using CRISPR/Cas9, which displayed the classic double muscling, characterised by hyperplasia and hypertrophy of muscle fibres. These animals exhibited a substantial increase in muscle mass and overall body weight compared with wild‐type counterparts, and the phenotype was stably inherited by subsequent generations. Similarly, Zhang, Lu, et al. (2019) and Zhang, Ran, et al. (2019) targeted the cystine‐knot motif in exon 3 of *MSTN* via CRISPR/Cas9, producing founder rabbits with 20%–30% higher body weights compared with wild‐type rabbits. Together, these results confirm the potential of *MSTN* disruption to markedly improve lean meat yield in rabbit production systems.

While *MSTN*‐knockout rabbits offer substantial gains in meat quantity, their impact on quality attributes requires careful evaluation. Sternstein et al. (2014) identified an *MSTN* SNP (c.373 + 234G>A) that was significantly associated with carcass composition traits, including skeletal muscle and bone weights, in a crossbred rabbit population. These findings suggest that *MSTN* variation influences not only growth but also the partitioning of carcass components. Kuang et al. (2014) observed breed‐related differences in *MSTN* expression and muscle fibre composition, with fast‐growing breeds exhibiting lower *MSTN* mRNA expression levels and altered proportions of oxidative and glycolytic fibres. These shifts in muscle fibre types are likely to influence tenderness, juiciness, and flavour. Although *MSTN*‐knockout rabbits have not yet been deeply evaluated for sensory traits, the observed muscle hypertrophy raises concerns about potential reductions in intramuscular fat and changes in connective tissue content, which could affect palatability. As in other livestock species, the sustainable use of *MSTN*‐driven genetic improvements in rabbit meat production will require balancing enhanced lean yield with the preservation of desirable quality traits.

### 1.5 The Role of Myostatin in Reproduction

Beyond its role in skeletal muscle development, *MSTN* has been implicated in reproductive physiology and embryonic development. Although mutations in *MSTN* offer clear productive advantages, they may cause secondary effects on the reproductive system that vary between species (Table 1). Moreover, the application of such genetic manipulations raises ethical and physiological considerations that require careful evaluation.

#### 1.5.1 Cattle

In Marchigiana, an Italian beef cattle breed, recent studies have shown that heterozygous bulls for the *MSTN* mutation (G/T) display significantly greater muscular development than wild‐type (G/G) individuals, with no major negative effects on semen quality. Higher ejaculate volumes have been reported in heterozygous bulls compared to wild‐type, although slight reductions in testicular diameter and scrotal circumference were observed (Colombi, Perini, et al. ). Similarly, Zhao et al. (2025) reported that *MSTN*‐edited Luxi cows had larger pelvic areas, a trait that could mitigate calving difficulties (dystocia) for calves with increased birth weight. Reproductive organ size in *MSTN*‐edited Luxi cows was comparable to that of wild‐type animals, and hormonal profiles indicated only subtle endocrine changes. These findings support the feasibility of incorporating certain *MSTN* mutations in breeding programs when appropriately managed. However, *MSTN* mutations can also have significant drawbacks. In breeds such as Belgian Blue and Piemontese, the pronounced muscularity is often accompanied by dystocia due to calves with greater weight and muscle volume, often requiring caesarean section (Hanset and Jandrain 1979). Ryan et al. (2023) found that some variants, such as *nt821*, *Q204X*, and *F94L*, were associated with increased calving difficulty when present in dams or progeny. Moreover, selection for inactive *MSTN* alleles has shown pleiotropic effects on reproduction, including reduced fertility and altered oestrous cycles in cows (Hoflack et al. 2006). Overall, while specific *MSTN* alleles may offer productive benefits, such as improved carcass traits and enhanced muscle development, others are linked to calving difficulties and compromised fertility. These contrasting outcomes highlight the importance of carefully selecting *MSTN* variants that balance muscling potential with reproductive performance, ensuring that breeding objectives do not inadvertently compromise animal welfare or reproductive efficiency.

#### 1.5.2 Sheep

Similarly to what has been observed in cattle, sheep carrying inactivating mutations in *MSTN*, such as those in the Texel breed, may exhibit pleiotropic effects, dystocia, and neonatal complications. The reproductive challenges are primarily due to the elevated birth weights of lambs, which can cause significant stress in ewes and often require obstetrical interventions (Hadjipavlou et al. 2008). Recent studies in gene‐edited sheep have provided additional insights into the potential reproductive effects of *MSTN* mutations. Chen et al. (2023) examined *MSTN* and *FGF5* dual‐gene knockout sheep and found that, despite the pronounced DBM phenotype, there were no significant differences in semen quality, sperm motility, or fertilisation rates between knockout (MF−/−) and wild‐type rams. Additionally, heterozygous MF+/− sheep showed no pathological alterations in ovarian histology or reproductive organ morphology compared to wild‐type animals. These findings suggest that *MSTN* mutations, when carefully managed, do not severely compromise reproductive performance in sheep. However, the authors note that monitoring is still essential because *MSTN* mutations can interact with other physiological systems, potentially influencing traits such as the spleen index or visceral organ morphology. Overall, these results highlight the need for balanced breeding strategies that optimise both muscle development and reproductive efficiency.

#### 1.5.3 Goats

In goats, CRISPR/Cas9 editing of *MSTN* has resulted in increased body weights at birth and at weaning in *MSTN*−/− kids (Kalds et al. 2023; Wang et al. 2018). Although direct data on kidding difficulties are limited, evidence from other livestock species (e.g., cattle dystocia) suggests potential risks that require species‐specific investigation. Therefore, given the effects observed in other livestock species, *MSTN* allele deployment in goat genetic improvement programs should proceed with caution. Targeted investigations focusing on reproductive efficiency, kidding performance, and maternal health in caprine livestock systems are essential to ensure that productivity gains do not compromise animal welfare and long‐term sustainability.

#### 1.5.4 Pigs

In pigs, the effect of *MSTN* mutations on reproduction has been well documented. Han et al. (2021) observed that heterozygous sows (*MSTN*+/−) exhibited delayed puberty and required a higher number of matings to achieve pregnancy. Although the heterozygous *MSTN*+/− sows retain normal natural reproductive capacity, their average litter size was significantly lower compared with that of wild‐type animals (7.75 ± 0.44 vs. 14.25 ± 0.60, respectively). Moreover, *MSTN*−/− piglets showed a higher incidence of congenital defects, such as macroglossia and umbilical hernias, along with an increased neonatal mortality rate. Han et al. (2019) in a recent study have demonstrated that homozygous myostatin‐knockout (*MSTN*−/−) boars exhibit a pronounced double‐muscling phenotype without detectable abnormalities in reproductive organs or semen quality, despite a reduced ejaculate volume compared to wild‐type controls. Matika et al. (2019) highlighted a premature stop mutation in *MSTN* associated with a recessive leg weakness syndrome in pigs. These findings highlight the need to carefully assess the reproductive impacts when incorporating *MSTN* mutations into pig breeding programs (Fan et al. 2022).

#### 1.5.5 Poultry

In chickens, MSTN inhibition enhances muscle development but may negatively influence reproductive performance. Lee et al. (2021b) reported a delayed onset of egg laying and reduced peak egg production in both quail and chickens carrying *MSTN* modifications. In broiler lines, modified *MSTN* animals showed increased egg weight alongside decreased hatchability and embryonic viability (Lee et al. 2024). *MSTN*‐knockout hens exhibited altered laying intervals and reduced clutch sizes (Lee et al. 2021a). High levels of MSTN expression detected in embryonic gonadal tissues also suggest direct effects on gametogenesis (Kubota et al. 2007). These trade‐offs highlight the need to balance muscle growth gains with reproductive efficiency in poultry breeding programs.

#### 1.5.6 Rabbits

In rabbits, CRISPR/Cas9 *MSTN*−/− animals exhibit pronounced muscle hypertrophy along with anatomical abnormalities, including tooth dislocation, macroglossia, and pelvic tilt (Zhang, Lu, et al. 2019; Zhang, Ran, et al. 2019). Although specific data on reproductive performance are currently limited, the observed phenotypic alterations highlight the pleiotropic effects of *MSTN* disruption. Such evidence underscores the need for comprehensive phenotypic evaluation, including reproductive traits, before deploying *MSTN* mutations in breeding programs.

### 1.6 The Role of Myostatin in Feed Conversion

Loss‐of‐function mutations in *MSTN* are known to induce marked muscle hypertrophy and have consistently been linked to enhanced FE and reduced feed conversion ratio (FCR) across livestock species. Animals carrying such mutations utilise nutrients more effectively, preferentially directing dietary energy towards lean tissue accretion rather than fat deposition. Consequently, they require less feed per unit of meat produced, resulting in both improved productivity and lower environmental impact. Accordingly, FE tends to increase, and FCR tends to decrease in animals harbouring *MSTN* mutations, confirming the central role of this gene in modulating growth performance and resource utilisation (Table 1).

Evidence from different species reinforces this general pattern.

In cattle, *MSTN* mutations have been shown to enhance FE and reduce total feed requirements, suggesting opportunities to improve growth rates while lowering the environmental footprint of beef production (Fiems 2012; Lee et al. 2021a).

Similar effects have been reported in poultry: *MSTN*‐mutant quail exhibit higher body weight gain and reduced FCR during the grow‐out period, with decreased fat deposition and more efficient conversion of dietary energy into muscle mass (Lee et al. 2021a). Conversely, increased *MSTN* expression under heat stress conditions has been associated with impaired FE in broilers, further highlighting its regulatory role under challenging production conditions (Li et al. 2021).

In pigs, *MSTN* editing leads to greater muscle mass; however, the resulting increase in maintenance energy expenditure may partially offset the benefits of reduced physical activity, leading to FCR values that are comparable to those of wild‐type animals. Moreover, the relatively limited natural variability in *MSTN* alleles in swine populations has constrained the extent of genetic improvement achievable through selection alone (Fan et al. 2022).

Similar findings have been observed in small ruminants. Sheep carrying *MSTN* mutations show improved FCR and leaner carcasses under high‐nutrition conditions (Haynes et al. 2012). Integrating *MSTN*‐based selection with additional molecular markers has been proposed as a promising approach to further enhance production efficiency and sustainability in the ovine sector (ChackoKaitholil et al. 2024). In goats, although research remains limited, gene‐edited *MSTN*‐knockout animals exhibit pronounced muscle hypertrophy, suggesting similar benefits on FE through a preferential allocation of nutrients toward protein accretion (Kalds et al. 2023).

Emerging evidence in rabbits highlights that MSTN modulation may hold promise within genomic selection and precision nutrition frameworks aimed at reducing production costs and improving FE, although direct empirical data on FE outcomes in this species remain limited (Goswami et al. 2025).

Taken together, findings across species converge on a consistent conclusion: suppression or mutation of *MSTN* can enhance feed conversion and efficiency, positioning this target as a strategic focus for future integrated genetic and nutritional strategies aimed at improving the environmental sustainability of livestock production systems and contributing to meeting the increasing global requirement for animal‐derived protein.

## 2 Conclusions and Future Perspectives

*MSTN* represents a central target in livestock genetic improvement for enhanced muscle growth, lean yield, and overall production efficiency. Across multiple species, *MSTN* mutations have demonstrated remarkable potential to improve growth traits and carcass characteristics. From an industry perspective, *MSTN* manipulation, whether via natural polymorphisms, selection, or gene‐editing tools, offers a unique opportunity to enhance meat production and production efficiency, integrating FE, growth kinetics, carcass traits, and resource‐use optimisation across livestock production systems. However, these interventions require a cautious, species‐specific approach that balances production gains with consumer expectations and ethical frameworks. The integration of *MSTN* into breeding programs should be carried out through multi‐trait genomic selection indices, considering correlated effects on reproduction, meat quality, and animal resilience. Future advances in functional genomics, genome editing, and precision breeding are expected to further expand the potential applications of *MSTN* in livestock improvement programs, facilitating the production of livestock‐derived foods that are both resource‐efficient and aligned with societal expectations regarding animal welfare and product quality. Achieving this vision will require a coordinated multidisciplinary approach that combines cutting‐edge science with responsible governance and active participation of all stakeholders involved in sustainable livestock systems, from producers to policymakers and consumer communities.

## Author Contributions

**Ishaku L. Haruna:** conceptualization, project administration, supervision, writing – review and editing. **Stefano Bettini:** writing – review and editing. **Daniele Colombi:** conceptualization, investigation, writing – review and editing. **Francesco Perini:** conceptualization, investigation, writing – review and editing. **Simone Ceccobelli:** writing – review and editing. **Marina Pasquini:** writing – review and editing. **Ketan Patel:** conceptualization, project administration, supervision, writing – review and editing. **Maria Federica Trombetta:** writing – review and editing. **Emiliano Lasagna:** conceptualization, project administration, supervision, writing – review and editing. **Chiara Santomassimo:** conceptualization, investigation, writing – original draft, writing – review and editing.

## Conflicts of Interest

The authors declare no conflicts of interest.

## Data Availability

Data sharing is not applicable to this article as no new data were generated or analysed in this study.
